# Comparison of postoperative acute kidney injury between ileal conduit and neobladder urinary diversions after radical cystectomy: A propensity score matching analysis

**DOI:** 10.1097/MD.0000000000004838

**Published:** 2016-09-09

**Authors:** Kyoung-Woon Joung, Yu-Gyeong Kong, Syn-Hae Yoon, Yeon Ju Kim, Jai-Hyun Hwang, Bumsik Hong, Young-Kug Kim

**Affiliations:** a Department of Anesthesiology and Pain Medicine; b Department of Urology, Asan Medical Center, University of Ulsan College of Medicine, Seoul, Republic of Korea.

**Keywords:** acute kidney injury, radical cystectomy, urinary diversion

## Abstract

Ileal conduit and neobladder urinary diversions are frequently performed after radical cystectomy. However, complications after radical cystectomy may be different according to the type of urinary diversion. Acute kidney injury (AKI) is a common complication after surgery and increases costs, morbidity, and mortality of hospitalized patients. This study was performed to compare the incidence of postoperative AKI between ileal conduit and neobladder urinary diversions after radical cystectomy.

All consecutive patients who underwent radical cystectomy in 2004 to 2014 in a single tertiary care center were identified. The patients were divided into the ileal conduit and ileal neobladder groups. Preoperative variables, including demographics, cancer-related data and laboratory values, as well as intraoperative data and postoperative outcomes, including AKI, intensive care unit admission rate, and the duration of hospital stay, were evaluated between the groups. Postoperative AKI was defined according to the Kidney Disease: Improving Global Outcome criteria. Propensity score matching analysis was performed to reduce the influence of possible confounding variables and adjust for intergroup differences.

After performing 1:1 propensity score matching, the ileal conduit and ileal neobladder groups each included 101 patients. The overall incidence of AKI after radical cystectomy was 30.7% (62 out of 202) and the incidences did not significantly differ between the groups (27 [26.7%], ileal conduit group vs 35 [34.7%], ileal neobladder group, *P* = 0.268). Intraoperative data, intensive care unit admission rate, and the duration of hospital stay were not significantly different between the groups.

Postoperative AKI did not significantly differ between ileal conduit and neobladder urinary diversions after radical cystectomy. This finding provides additional information useful for appropriate selection of the urinary diversion type in conjunction with radical cystectomy.

## 1 Introduction

Radical cystectomy with urinary diversion is the standard treatment for high-grade muscle invasive bladder cancer. Various types of urinary diversion, including ileal conduit and ileal neobladder, have been performed after radical cystectomy. Ileal conduit urinary diversion is considered to be a standard diversion procedure because it is regarded as the most clinically adequate, reliable, and cost-effective solution for bladder cancer.^[1,2]^ On the other hand, ileal neobladder is the most commonly used urinary diversion method because it is associated with a better quality of life.^[3]^

Complications after radical cystectomy may be different according to the type of urinary diversion. Paralytic ileus and stoma-associated complications are known to be the most common early and late complications in ileal conduit urinary diversion.^[1]^ In contrast, metabolic acidosis and hydronephrosis commonly occur in ileal neobladder urinary diversion.^[4]^ Because there are inherent problems with each form of urinary diversion, the type used in conjunction with radical cystectomy should be carefully selected. Especially, patient characteristics and postoperative complications should be considered when making the selection.

Acute kidney injury (AKI) is an important complication after radical cystectomy because it is associated with the cost and morbidity of hospitalized patients.^[5,6]^ Moreover, AKI after radical cystectomy is also known to be associated with a higher incidence of chronic kidney disease and mortality.^[7]^ Recently, the overall incidence of AKI after radical cystectomy was reported to be 31% to 38%;^[7,8]^ however, little is known regarding the incidences of AKI for the different types of urinary diversion. Therefore, we compared the incidence of postoperative AKI between ileal conduit and neobladder urinary diversions after radical cystectomy. We also compared postoperative outcomes between the 2 types of urinary diversion.

## 2 Methods

### 2.1 Study population

After approval from the Institutional Review Board (2016–0096) of our institution, the electronic medical records for patients with muscle-invasive bladder cancer who underwent radical cystectomy at Asan Medical Center, Seoul, Republic of Korea, between 2004 and 2014 were reviewed. We excluded patients who met the following criteria: patients <20 years old, patients who underwent additional procedures with radical cystectomy, patients with a history of chronic kidney disease, and patients with incomplete perioperative data. We reported this retrospective observational study according to the Strengthening the Reporting of Observational Studies in Epidemiology guidelines.^[9]^

### 2.2 Anesthetic and surgical technique

As previously described,^[8]^ anesthetic techniques for radical cystectomy were performed as per our institutional standards. General anesthesia was induced using thiopental or propofol, fentanyl, and muscle relaxants (vecuronium or rocuronium) and was maintained using volatile anesthetics (isoflurane, sevoflurane, or desflurane), a 50% O_2_/air mixture, fentanyl, and muscle relaxants (vecuronium or rocuronium). Crystalloid and colloid solutions (6% hydroxyethyl starch 130/0.4) were administered during surgery. Arterial blood pressure during anesthesia was maintained at >65 mm Hg of mean arterial pressure or >90 mm Hg of systolic arterial pressure. Packed red blood cell transfusion was performed during the perioperative period when the hemoglobin concentration was <8 g/dL.

Details of the surgical techniques have been previously described.^[10]^ Radical cystectomy and pelvic lymphadenectomy were performed by standard techniques at our institution. The extent of lymph node dissection ranged from standard lymphadenectomy, including the distal common iliac, external iliac, hypogastric, obturator, and perivesical lymph nodes, to extended lymphadenectomy, including the mentioned lymph nodes plus those at the level of the proximal common iliac artery, distal aorta, and vena cava. Subsequently, urinary diversions, including ileal conduit and ileal neobladder types, were performed. In all cases, the surgeon and patient together decided on the type of urinary diversion that would be used. The ureteral stents were inserted at the end of ureter anastomosis to prevent transient obstruction on some patients according to surgeons’ decision, and the removal of the stents was done 1 to 2 months after the surgery.

In addition, neoadjuvant chemotherapy was performed on some patients, according to surgeons’ and oncologists’ decision. The regimens of neoadjuvant chemotherapy were combination of gemcitabine/carboplantin or gemcitabine/cis-dichlorodiammineplatinum.

### 2.3 Measurements and definitions

The collected data included demographics (sex, age, height, weight, body mass index, and comorbidity, such as hypertension, diabetes mellitus, coronary artery disease, and cerebrovascular disease), cancer-related data (cancer stage and grade), neoadjuvant chemotherapy application, medications, laboratory values (serum hematocrit, albumin, creatinine, and uric acid), ureteral stent placement, and intraoperative data (operation time, infused crystalloid and colloid amounts, blood transfusion rate, and use of vasoactive drugs). Hypertension was defined as systolic blood pressure >140 mm Hg, diastolic blood pressure >90 mm Hg, or medication with an antihypertensive drug. Coronary artery disease was defined as a history of ischemic heart disease diagnosed by a cardiologist. Cerebrovascular disease was defined as a history of carotid artery stent or angioplasty, transient ischemic attack, stroke, or cerebral hemorrhagic event. Cancer stage and grade were confirmed by genitourinary pathologists. A cancer stage was assigned according to the 2010 American Joint Committee on Cancer tumor–node–metastasis staging system,^[11]^ and cancer grade was assigned according to the 2004 WHO grading system.^[4]^

### 2.4 Primary and secondary outcomes

The primary outcomes included the incidences of AKI between the ileal conduit and ileal neobladder groups. Postoperative AKI after radical cystectomy was defined according to Kidney Disease: Improving Global Outcome (KDIGO) criteria.^[12]^ According to the KDIGO criteria, AKI is defined as an increase in serum creatinine by ≥0.3 mg/dL within 48 hours or a ≥1.5-fold increase in serum creatinine within the previous 7 days. The urine output criterion of KDIGO was not used. The secondary outcomes were the postoperative outcomes including intensive care unit admission rate and the duration of hospital stay between the 2 groups.

### 2.5 Statistical analysis

All continuous variables were expressed as mean ± SD. Categorical variables were expressed as number (%). Before propensity matching, we compared demographics, cancer-related data, medications, and preoperative laboratory values between the ileal conduit and ileal neobladder groups using the *t* test or Mann–Whitney *U* test for continuous variables and the Chi-square test or Fisher exact test for categorical variables as appropriate. To reduce the influence of confounding variables, we performed 1:1 propensity score matching analysis. The propensity score was determined with multiple logistic regression using the following variables: sex; age; height; weight; body mass index; history of hypertension, diabetes mellitus, coronary artery disease, or cerebrovascular disease; cancer stage and grade; neoadjuvant chemotherapy application; angiotensin-converting enzyme inhibitor, angiotensin II receptor blocker, or diuretic medications; hematocrit; albumin; creatinine; uric acid; and ureteral stent placement (Table 1). After performing 1:1 propensity score matching, we compared intraoperative data and postoperative outcomes, including AKI, intensive care unit admission rate, and the duration of hospital stay, between the 2 groups using the paired *t* test or Wilcoxon signed-rank test for continuous variables and the McNemar test for categorical variables as appropriate. All reported *P* values were 2-sided and *P* values <0.05 were considered to indicate statistical significance. All data manipulations and statistical analyses were performed by using SPSS Version 21.0 software (IBM Corp, Armonk, NY).

**Table 1:**
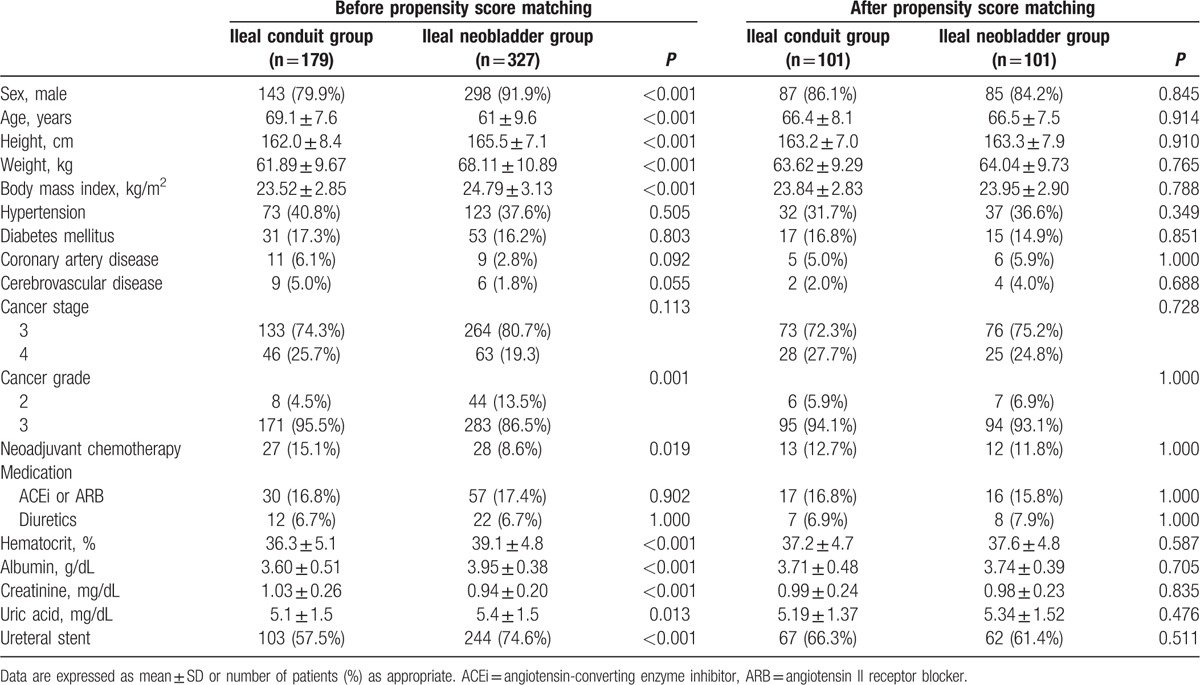
Variables of propensity score matching analysis.

## 3 Results

**Figure 1:**
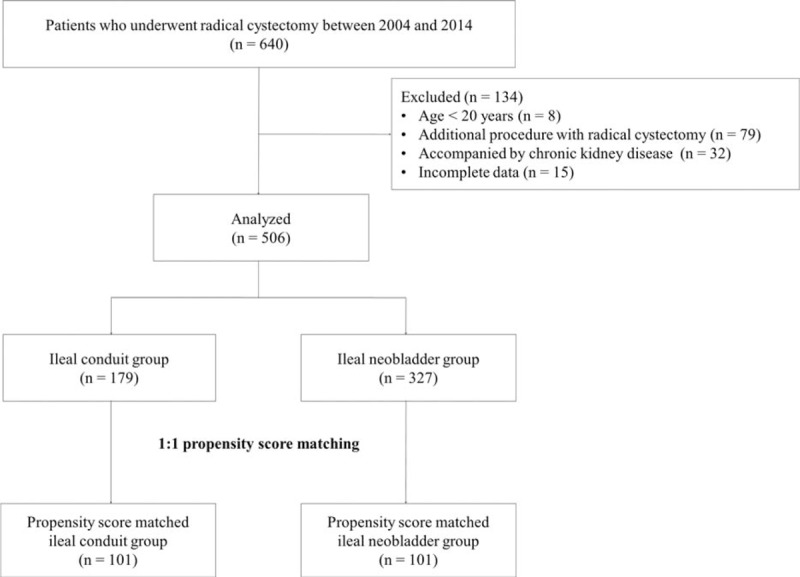
Study flow diagram.

The medical chart review identified 640 patients who underwent radical cystectomy during the study period. Of these, 134 patients were excluded because they were <20 years old (n = 8), underwent additional procedures to radical cystectomy (n = 79), had a history of chronic kidney disease (n = 32), or had incomplete perioperative data (n = 15) (Fig. 1). Of the remaining 506 patients, 179 underwent ileal conduit urinary diversion and 327 underwent ileal neobladder urinary diversion after radical cystectomy. The variables including sex, age, height, weight, body mass index, cancer grade, neoadjuvant chemotherapy application, hematocrit, albumin, creatinine, uric acid levels, and ureteral stent placement were significantly different between the 2 groups. After propensity matching, demographics, cancer-related data, neoadjuvant chemotherapy application, medications, preoperative laboratory data, and ureteral stent placement were not significantly different between the ileal conduit (n = 101) and ileal neobladder groups (n = 101) (Table 1).

The variables including operation time, infused crystalloid and colloid amounts, blood transfusion rate, and use of vasoactive drugs were not significantly different between the 2 groups (Table 2). The overall incidence of AKI after radical cystectomy was 30.7% (62 out of 202), and the respective differences were not significantly different between the 2 groups (27 [26.7%] in the ileal conduit group vs 35 [34.7%] in the ileal neobladder group; *P* = 0.268). Intensive care unit admission rate and the duration of hospital stay were not significantly different between the 2 groups (Table 2).

**Table 2:**
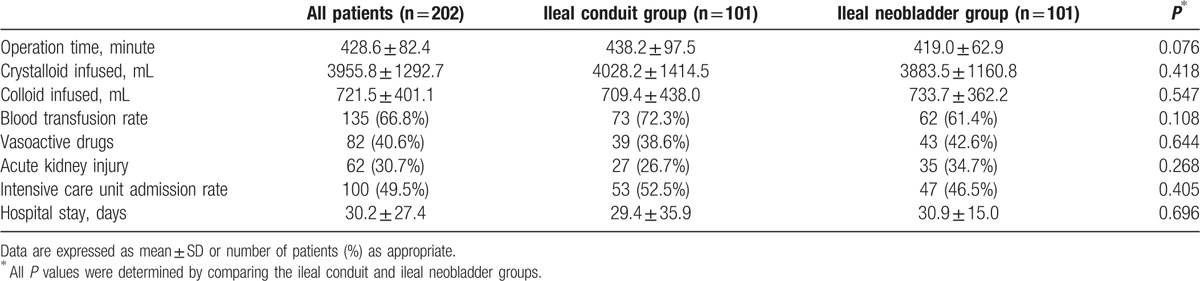
Intraoperative data and postoperative outcomes for propensity score matched patients who underwent ileal conduit or ileal neobladder urinary diversion.

## 4 Discussion

This study showed that 30.7% of the patients who underwent radical cystectomy developed postoperative AKI, and the incidences did not significantly differ between the ileal conduit and ileal neobladder groups.

In muscle invasive bladder cancer, radical cystoprostatectomy in male patients and anterior pelvic exenteration in female patients coupled with en-bloc pelvic lymphadenectomy remains the standard surgical treatment. Prior to the 1950s, the urinary diversion of choice was ureterosigmoidostomy, which used the anal sphincter. After Bricker report,^[13]^ bowel conduits were considered the gold standard for urinary diversion until recently. However, bowel conduits have some disadvantages, such as external ostomy-associated negative body image, stomal stenosis, and parastomal hernia.^[14,15]^ Thus, the neobladder technique was reintroduced in 1979,^[16]^ and other investigators have improved this technique.^[17]^ A technique that would offer the greatest advantage would be one that has no external devices for urination and preserves anatomical voiding. Therefore, the ileal neobladder urinary diversion provides better quality of life than the ileal conduit urinary diversion.^[18,19]^ On the other hand, ileal neobladder urinary diversion is technically more difficult and is associated with more frequent nocturnal incontinence than ileal conduit urinary diversion.^[14,15]^ Considering the pros and cons of each procedure, there is no single technique that is ideal for all patients and clinical situations. Thus, the type of urinary diversion in conjunction with radical cystectomy should be carefully selected after considering various conditions, such as functional outcomes, postoperative complications, and physical and psychosocial factors.^[14,15]^

Although complications after radical cystectomy may be different according to the type of urinary diversion, the overall incidence of postoperative complications are similar. For example, Parekh et al^[20]^ showed that incidences of postoperative complications such as ileus, fistula, and deep vein thrombosis were not different between the ileal conduit and ileal neobladder groups. Furthermore, Sogni et al^[21]^ reported that morbidity and mortality were not different between the 2 types of surgeries.

Postoperative AKI is one of the most critical complications after surgery because it increases costs, morbidity, and mortality.^[6,7]^ However, no studies have compared the incidences of postoperative AKI between ileal conduit and neobladder urinary diversions in conjunction with radical cystectomy. In the present study, we found that there was no significant difference in the incidences of postoperative AKI between the 2 groups. This result could be explained by the following: first, there was no difference in the surgical time between the ileal conduit and ileal neobladder groups. Many studies have suggested that prolonged surgical time is associated with postoperative AKI. For example, Licker et al^[22]^ reported that prolonged operation time was associated with a risk of AKI after lung cancer surgery. Our previous study showed that long surgical time was an independent risk factor for AKI after radical cystectomy.^[8]^ Second, there was no difference in blood transfusion requirement, which is another well-known risk factor of AKI after surgery, between the 2 groups. A recent retrospective study showed that intraoperative blood transfusion was associated with AKI after major abdominal surgery.^[23]^ Other studies have also demonstrated that blood transfusion was associated with AKI development under various conditions, such as postcardiac surgery and septic shock condition.^[24,25]^ Last, the amount of fluid administered, especially colloid, was also not different between the 2 groups. Lee et al^[26]^ demonstrated that colloid infusion during surgery was an independent risk factor for AKI after esophageal cancer surgery. More recently, Hand et al^[27]^ also reported that colloid administration was associated with AKI development after liver transplantation.

An interesting consideration related to our findings is that we showed the incidence of AKI after radical cystectomy according to KDIGO criteria. The Risk, Injury, Failure, Loss, End-Stage (RIFLE) criteria were first created to standardize the definition and classification of AKI.^[28]^ However, these criteria were not designed to predict mortality or adverse outcomes. Thus, to make the RIFLE criteria more sensitive and reliable, nephrologists and intensivists have modified the RIFLE criteria and published them as the Acute Kidney Injury Network (AKIN) criteria.^[29]^ More recently, the KDIGO Acute Kidney Injury Work Group modified the AKIN criteria. These have been important novel criteria in medical practice, especially with regard to the criterion of time. The KDIGO criteria cover both the AKIN and RIFLE criteria and take into account changes in creatinine within 48 hours or a decline in the glomerular filtration rate over 7 days. In this context, the KDIGO criteria are the most sensitive criteria for the detection of AKI.

The present observational study had some unavoidable limitations. There were many confounders, such as sex, age, body mass index, comorbidity, and preoperative laboratory examination, which may have affected AKI development after radical cystectomy. Although we performed propensity score matching analysis for 19 confounding factors to minimize these biases, we cannot exclude the possibility of another confounder that may have influenced the outcomes. However, there are many difficult problems to overcome in a well-designed randomized controlled trial to compare ileal conduit and neobladder urinary diversions. Especially, most patients do not wish to be randomized to a specific surgical treatment. Thus, we think that the propensity score matching analysis can be a reliable 2nd-best strategy for comparing ileal conduit and neobladder urinary diversions.

In conclusion, the incidence of postoperative AKI did not differ between ileal conduit and neobladder urinary diversions after radical cystectomy. These results provide additional information useful for optimal selection of the suitable type of urinary diversion performed in conjunction with radical cystectomy.
